# Supplementary material for: Genome editing in butterflies reveals that spalt promotes and Distal-less represses eyespot colour patterns
Source: Nat Commun. 2016 Jun 15;7:11769. doi: 10.1038/ncomms11769 (PMC4912622; doi:10.1038/ncomms11769)
Supplement: Supplementary Information — Supplementary Figures 1-2 and Supplementary Table 1 [file ncomms11769-s1.pdf]

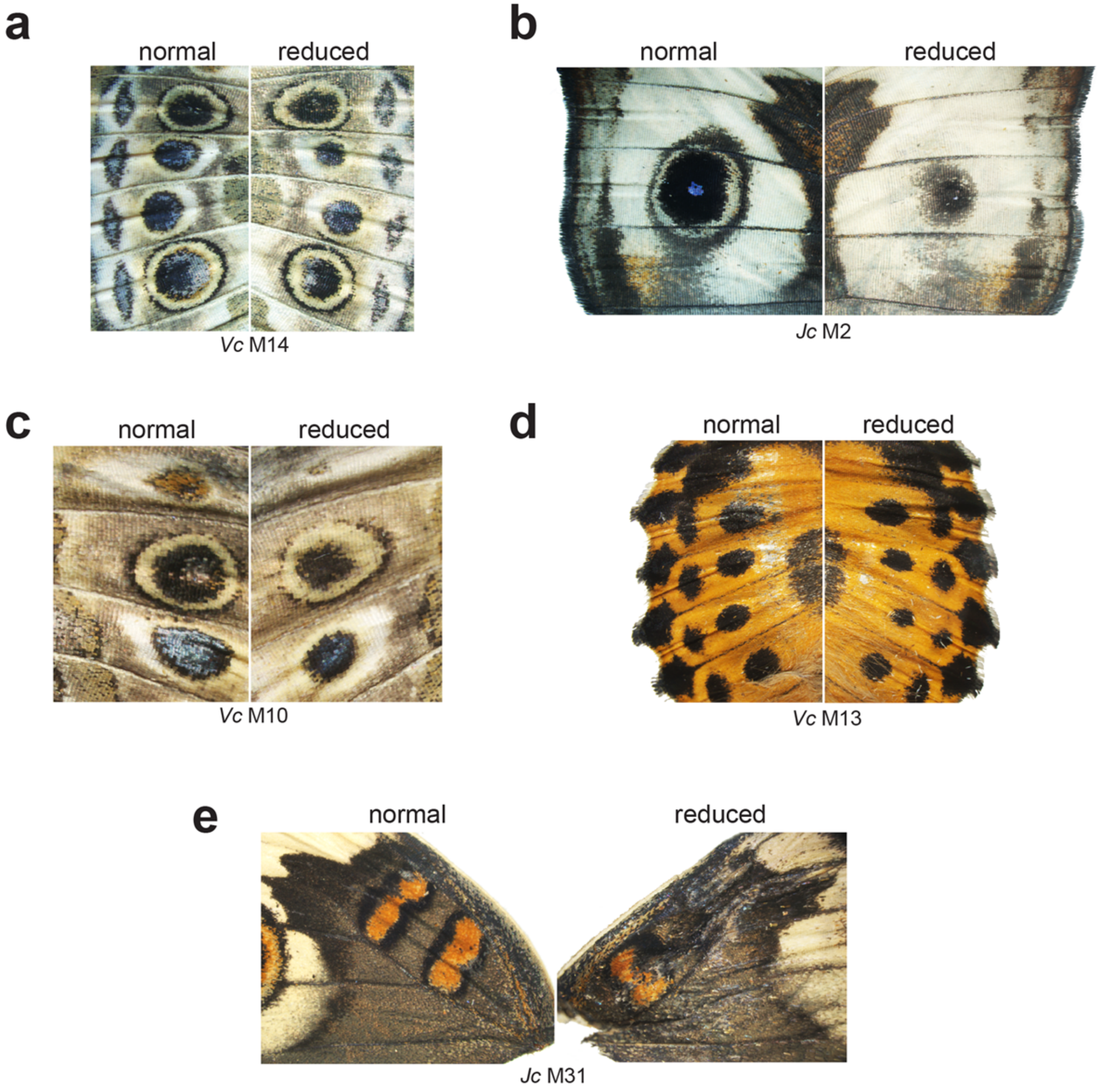

Supplementary Figure 1: Additional examples of color pattern reduction caused by *spalt* deletions. Examples of minor eyespot reduction phenotypes: (**a**) *V. cardui* ventral hindwing; (**b**) *J. coenia* ventral forewing; (**c**) *V. cardui* ventral hindwing; (**d**) *V. cardui* dorsal hindwing. (**e**) Loss of discal spot on a *J. coenia* dorsal forewing associated with vein abnormalities.

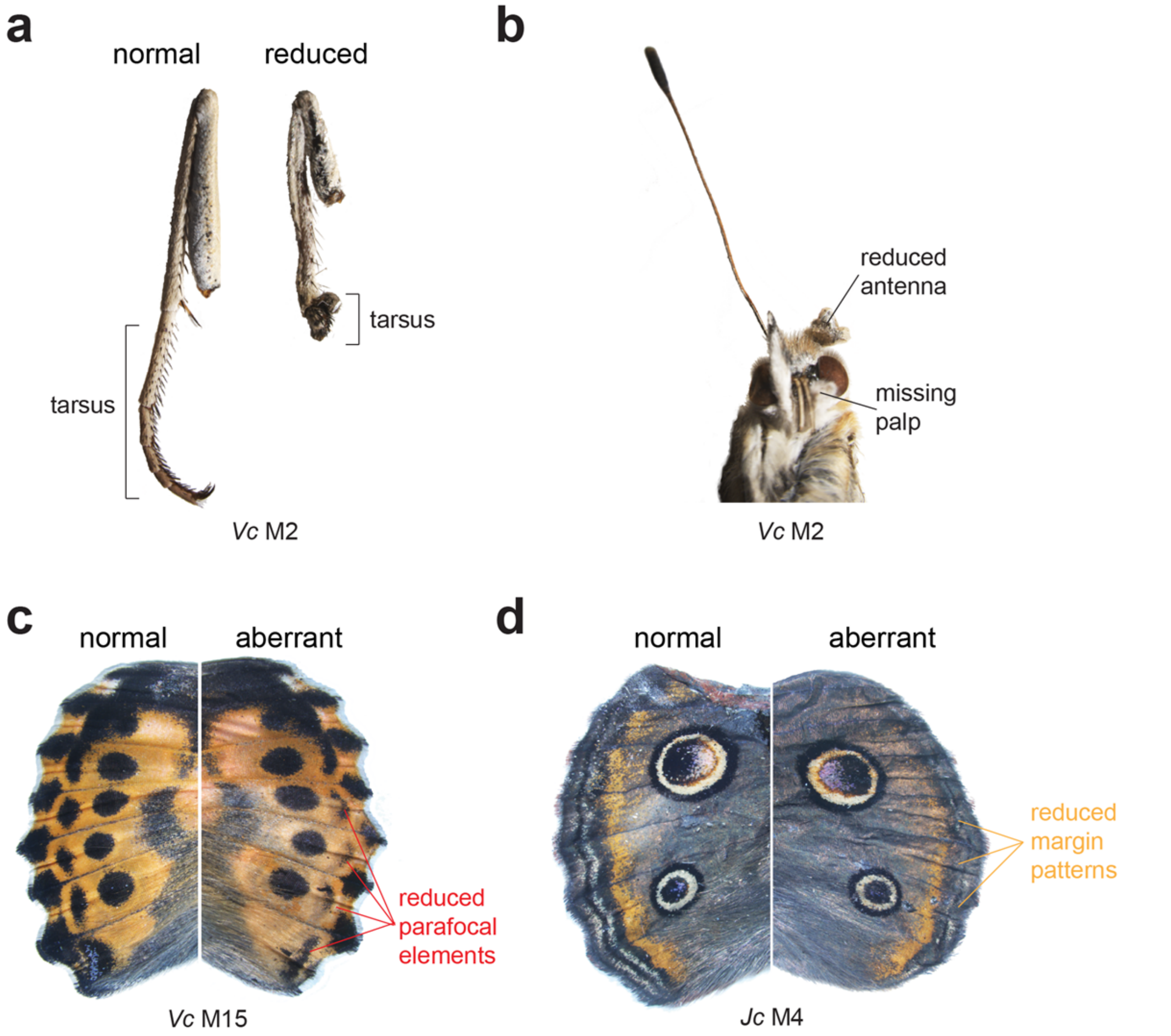

Supplementary Figure 2: Additional examples of *Dll* deletion phenotypes. (**a**) Reduction of the distal leg (tarsus) in *V. cardui*. (**b**) Bilateral reduction of antenna and palp in *V. cardui*. (**c**) Reduction of parafocal elements on *V. cardui* dorsal hindwing. (**d**) Reduction of margin patterns on dorsal *J. coenia* hindwing.

Supplementary Table 1: sgRNAs and primers used in this study

Target gene	sgRNA name	sgRNA sequence*	Genotyping primers
V. cardui	Vcddc_sgRNA1	GAAATTAATACGACTCACTATA**GGAGTACCGTTACCTGATGA**GTTTTA GAGCTAGAAATAGC	5′-GCTGGATCAGCTATCGTCT
Ddc	Vcddc_sgRNA2	GAAATTAATACGACTCACTATA**GGTGGTCGTGTTTCAAGTAGAG**GTTTTA GAGCTAGAAATAGC	5′-GCAGTAGCCTTTACTTCCTCCCAG
V. cardui	Vcspalt_sgRNA1	GAAATTAATACGACTCACTATA**GGACCAATTTCAATAGCAAC**GTTTTAGAGCTAGAAATAGC	5′-AGCAACGAAGATCAAATTGGCAGA
spalt	Vcspalt_sgRNA2	GAAATTAATACGACTCACTATA**GGGGAATATTGGCGGAGGGA**GTTTTAGAGCTAGAAATAGC	5′-ATGGGAGGGAAGCCGTTGTG
V. cardui	Vcdll_sgRNA1	GAAATTAATACGACTCACTATA**GGGCCTCCTCAGTCCTCGGA**GTTTTAGAGCTAGAAATAGC	5′-GTGATAGTGATGCTGTGTACTGCAGTT
Distal-less	Vcdll_sgRNA2	GAAATTAATACGACTCACTATA**GGAGAATCCTCATGAAGCCTCG**GTTTTAGAGCTAGAAATAGC	5′-CGCACTGTGGAGCATAGGAACCT
J. coenia	Jcspalt_sgRNA1	GAAATTAATACGACTCACTATA**GGAACGTTTAGAACAAGTGAGG**GTTTTAGAGCTAGAAATAGC	5′-CAATGCAAATGGGAGAAGAACG
spalt	Jcspalt_sgRNA2	GAAATTAATACGACTCACTATA**GGTGGGTCTTCCCTTTCCCG**GTTTTAGAGCTAGAAATAGC	5′-GAATTGTGCTACGGCAACTTTTGT
J. coenia	Jcdll_sgRNA1	GAAATTAATACGACTCACTATA**GGGCCTCCTCAGTCCTCGGA**GTTTTAGAGCTAGAAATAGC	5′-TGCGGGGCATAGGAACCTAAATG
Distal-less	Jcdll_sgRNA2	GAAATTAATACGACTCACTATA**GGAAACCCTGATGCCTCGTG**GTTTTAGAGCTAGAAATAGC	5′-TTACCCGAATTCAATCCCCTAGTAC

17 *: sgRNAs designed with GGN18NGG rule are marked with red GG; sgRNAs designed with N20NGG rule are marked with blue GG.
